# Intracranial Hemorrhage in Patients with Anticoagulant Therapy Undergoing Stereotactic Radiosurgery for Brain Metastases: A Bi-Institutional Analysis

**DOI:** 10.3390/cancers14030465

**Published:** 2022-01-18

**Authors:** Felix Ehret, David Kaul, Lucas Mose, Volker Budach, Peter Vajkoczy, Christoph Fürweger, Alfred Haidenberger, Alexander Muacevic, Felix Mehrhof, Markus Kufeld

**Affiliations:** 1 Berlin Institute of Health at Charité—Universitätsmedizin Berlin, 10117 Berlin, Germany; 2 Charité—Universitätsmedizin Berlin, Corporate Member of Freie Universität Berlin and Humboldt-Universität zu Berlin, Department of Radiation Oncology, 13353 Berlin, Germany; david.kaul@charite.de (D.K.); volker.budach@charite.de (V.B.); felix.mehrhof@charite.de (F.M.); 3 European Radiosurgery Center, 81377 Munich, Germany; l.mose@icloud.com (L.M.); christoph.fuerweger@erc-munich.com (C.F.); alfred.haidenberger@erc-munich.com (A.H.); alexander.muacevic@erc-munich.com (A.M.); markus.kufeld@erc-munich.com (M.K.); 4 Charité—Universitätsmedizin Berlin, Corporate Member of Freie Universität Berlin and Humboldt-Universität zu Berlin, Charité CyberKnife Center, 13353 Berlin, Germany; peter.vajkoczy@charite.de; 5 Charité—Universitätsmedizin Berlin, Corporate Member of Freie Universität Berlin and Humboldt-Universität zu Berlin, Department of Neurosurgery, 10117 Berlin, Germany; 6 Department of Stereotaxy and Functional Neurosurgery, University Hospital Cologne, 50937 Cologne, Germany

**Keywords:** brain metastases, intracranial hemorrhage, intratumoral hemorrhage, anticoagulant therapy, anticoagulation, stereotactic radiosurgery, CyberKnife

## Abstract

**Simple Summary:**

Patients suffering from cancer are at risk of various comorbidities that may require therapeutic anticoagulant therapy (ACT). Brain metastases (BM) are a common sequela of late-stage cancer and are often non-invasively treated with stereotactic radiosurgery (SRS). Although brain tumors and BM may cause intracranial hemorrhage (ICH), with potentially devastating consequences, the actual risk of bleeding complications for patients receiving ACT while undergoing SRS is mostly unknown. This bi-institutional analysis aimed to assess the incidence of ICH after SRS in patients with ACT. The results showed that ICHs mostly occurred in patients with BM originating from malignant melanomas and in those that showed signs of ICH before SRS treatment. In general, the bleeding events did not cause morbidity or mortality, suggesting the relatively safe use of ACT in patients with small- to medium-sized BM. Nevertheless, further studies are necessary to validate our reported findings.

**Abstract:**

Background: Stereotactic radiosurgery (SRS) is a well-established treatment modality for brain metastases (BM). Given the manifold implications of metastatic cancer on the body, affected patients have an increased risk of comorbidities, such as atrial fibrillation (AF) and venous thromboembolism (VTE), which includes pulmonary embolism (PE) and deep-vein thrombosis (DVT). These may require therapeutic anticoagulant therapy (ACT). Limited data are available on the risk of intracranial hemorrhage (ICH) after SRS for patients with BM who are receiving ACT. This bi-institutional analysis aimed to describe the bleeding risk for this patient subgroup. Methods: Patients with ACT at the time of single-fraction SRS for BM from two institutions were eligible for analysis. The cumulative incidence of ICH with death as a competing event was assessed during follow-up with magnetic resonance imaging or computed tomography. Results: Forty-one patients with 97 BM were included in the analyses. The median follow-up was 8.2 months (range: 1.7–77.5 months). The median and mean BM volumes were 0.47 and 1.19 cubic centimeters, respectively. The most common reasons for ACT were PE (41%), AF (34%), and DVT (7%). The ACT was mostly performed utilizing phenprocoumon (37%), novel oral anticoagulants (32%), or low-molecular-weight heparin (20%). Nine BM from a group of five patients with ICH after SRS were identified: none of them caused neurological or any other deficits. The 6-, 12-, and 18-month cumulative bleeding incidences per metastasis were 2.1%, 12.4%, and 12.4%, respectively. The metastases with previous bleeding events and those originating from malignant melanomas were found to more frequently demonstrate ICH after SRS (*p* = 0.02, *p* = 0.01). No surgical or medical intervention was necessary for ICH management, and no observed death was associated with an ICH. Conclusion: Patients receiving an ACT and single-fraction SRS for small- to medium-sized BM did not seem to have a clinically relevant risk of ICH. Previous bleeding and metastases originating from a malignant melanoma may favor bleeding events after SRS. Further studies are needed to validate our reported findings.

## 1. Introduction

Brain metastases (BM) represent a considerable medical and socioeconomic challenge and burden. An estimated 10 to 20% of patients with solid tumors will develop BM at some point during their disease course, accounting for more than 160,000 patients a year in the United States alone [1,2,3,4,5]. Despite recent advancements regarding targeted therapies and immunotherapies, BM bear a dismal overall prognosis [1]. With the increasing availability and technical refinements, stereotactic radiosurgery (SRS) has become an efficient treatment modality for patients with a limited number of BM [1,6]. Overall, SRS is considered a safe treatment option with a favorable risk and safety profile. Adverse events (AE) occur in less than 3% of treated patients [6,7]. The irradiation-induced complications may include motor dysfunctions, decreased neurocognitive function, visual field defects, headaches, nausea, and dizziness due to edema and its respective mass effects [7]. Intracranial hemorrhage (ICH) is believed to be a rare but potentially devastating side effect following SRS for malignant intracranial lesions, e.g., BM. ICH has also been described after the SRS of benign tumors, including meningiomas and schwannomas [8,9,10,11,12,13,14,15,16]. Preclinical studies demonstrated that endothelial cell damage leading to apoptosis is an early effect after the application of large single doses of radiation [17]. In the brain, endothelial damage may lead to the disruption of the blood–brain barrier and cause late-onset vascular effects, resulting in ischemic stroke and the formation of cavernoma or lacunar lesions. ICH, another serious consequence of radiation-induced endothelial damage, seems to occur at comparably low rates and is mostly dependent on the location of the underlying tumor or metastatic lesion, the dose, and the concomitant chemotherapy [18]. Another pathophysiological mechanism that may contribute to ICH after SRS is an increased intravascular outflow resistance in the residual neoplastic tissue, which leads to venous blood congestion and therefore promotes hemorrhage, an effect that could be more frequent in vessel-rich tumors [18,19,20]. An ICH in patients receiving therapeutic anticoagulant therapy (ACT) can result in fatal outcomes and significant morbidity [21,22]. Patients with metastatic brain lesions regularly show comorbidities such as atrial fibrillation (AF) and venous thromboembolism (VTE), which includes pulmonary embolism (PE) and deep-vein thrombosis (DVT) [23,24,25,26,27]. Thus, a considerable number of affected patients require ACT according to the respective guidelines [28]. In fact, the rate of VTE has been reported to be 4 to 6.5 times higher in metastatic cancer patients [29]. Cancer itself may also cause AF through a myriad of associated systemic factors such as inflammation, hypercoagulability, and autonomous dysregulation [27]. Several studies found a higher rate of AF in cancer patients and ICH rates of up to 40–50% in patients with BM receiving ACT [24,25,26,30,31]. BM can frequently show signs of hemorrhage in the absence of ACT depending on the tumor histology [32]. However, there is a dearth of reports investigating the outcomes concerning ICH in patients undergoing SRS for BM while receiving ACT. Applications of high doses of radiation energy—as typically used in SRS—might promote the risk of bleeding by interfering with the endothelial microenvironment and may trigger subsequent hemorrhage-inducing effects [18]. Given the potential devastating consequences of ICH, more analyses of this matter are indicated to determine the safety profile of ACT in SRS patients. Moreover, generally accepted guidelines regarding ACT for this specific patient group are lacking. Herein, we report our bi-institutional experience of the local bleeding complications after SRS for patients with BM receiving ACT.

## 2. Materials and Methods

Patients who were treated with single-fraction SRS for BM while receiving ACT at two institutions between 2005 and 2021 were eligible for analysis. ACT was performed either with phenprocoumon, novel oral anticoagulants (NOAC), synthetic heparin, or low-molecular-weight heparin (LMWH) at the time of SRS and—if deemed necessary—was controlled with the international normalized ratio (INR). SRS was performed using the Cyberknife^®^ robotic radiosurgery system (Accuray Inc., Sunnyvale, CA, USA) as previously described [33]. Treatment planning was based on computed tomography (CT) scans with 1 or 1.25 mm slice thickness. CT datasets were fused with magnetic resonance imaging (MRI) scans for target definition and the delineation of organs at risk (OAR). The dose prescription was standardized but adapted if deemed necessary due to the tumor size, the previous therapies, and the OAR. Patients with BM who received multiple pretreatments before SRS were included. Patient information and follow-up data, including pretreatments, tumor entity, imaging data, and survival data, were assessed and obtained through the electronic health records and patient files. Patients underwent regular clinical assessments and imaging investigations by CT or MRI. Only patients with at least one MRI scan after SRS were included herein. The occurrence of ICHs was assessed based on the evaluation of MRI scans. ICH was defined as signs of bleeding within or at the margin of the planning target volume (PTV) on an MRI scan, and was diagnosed by a board-certified radiologist or neuroradiologist. ICHs with and without clinical symptoms were considered. The primary endpoint was the cumulative incidence of post-SRS ICH and calculated on a per lesion basis. The secondary endpoints included the overall survival (OS) and the cumulative bleeding incidence per patient until the first ICH. OS was analyzed per patient from the date of the first SRS to the date of death by any cause or last patient contact. For OS analysis, the Kaplan–Meier estimate was applied. The bleeding incidences were analyzed per patient and per lesion from the date of SRS to the date of bleeding or last available radiographic follow-up. To account for the competing event of death for ICH, the cumulative bleeding incidence functions were assessed. For analysis of the subdistribution hazard of previous bleeding and pretreatments, a Fine and Gray competing risk regression model was utilized. Assessments of differences in time-to-event items were conducted utilizing the log-rank test. For categorical comparisons, Fisher’s exact test was applied. All *p*-values were two sided, with an α-level of 0.05. Statistical analyses were performed with STATA MP 16.0 (StataCorp, College Station, TX, USA).

## 3. Results

### 3.1. Patient and Treatment Characteristics

A total of 41 patients with 97 BM were identified and included in this analysis. The median age at treatment was 69.0 years, with the majority of the patients being male (23 patients, 56%). The median Eastern Cooperative Oncology Group (ECOG) performance status was 1. The most common reasons for receiving ACT were PE (17 patients, 41%), AF (14 patients, 34%), and DVT (3 patients, 7%). The ACT was mostly performed with phenprocoumon (15 patients, 37%), NOAC (13 patients, 32%), LMWH (8 patients, 20%), or synthetic heparin (3 patients, 7%). The underlying malignancies mostly comprised lung cancer (20 patients, 49%), malignant melanoma (6 patients, 15%), and renal cell carcinoma (5 patients, 12%). Most of the BM were located in the frontal lobe (35 BM, 36%), parietal lobe (19 BM, 20%), occipital lobe (16 BM, 16%), and cerebellum (10 BM, 10%). The median and mean BM sizes were 0.47 and 1.19 cubic centimeters (cc), respectively. Eight lesions in a group of six patients bled before these patients underwent SRS. Three patients each with one BM were previously treated with surgical metastasis resection; five patients had previously received whole-brain radiotherapy (WBRT). All of the patients underwent single-fraction SRS for their BM. The median dose and prescription isodose line were 20 Gray (Gy) and 70%, respectively. Sixteen patients (39%) were treated for multiple BM. The patient and treatment characteristics are summarized in Table 1.

### 3.2. Treatment Outcomes

**Figure 1:**
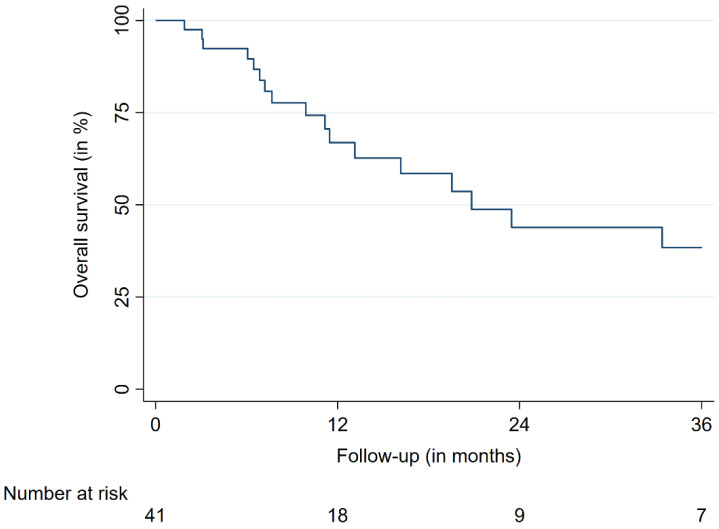
Overall survival.

**Figure 2:**
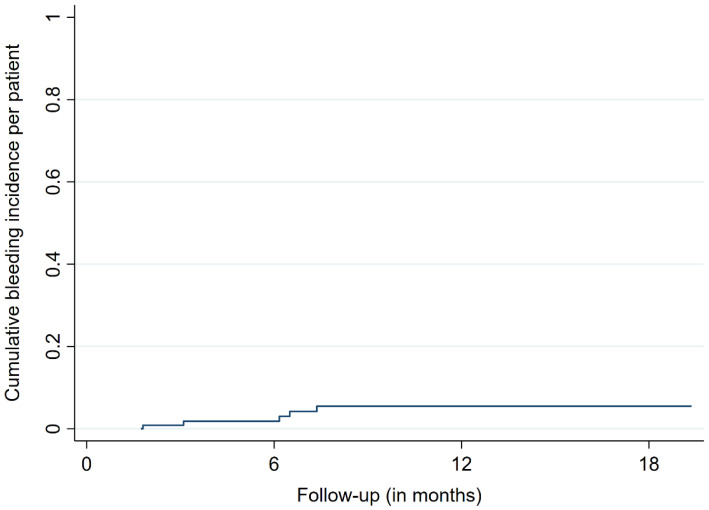
Cumulative incidence of bleeding events per patient.

**Figure 3:**
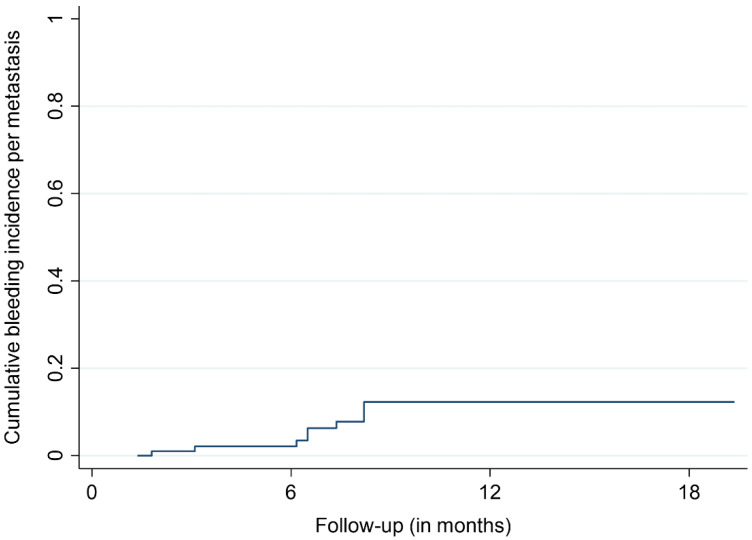
Cumulative incidence of bleeding events per metastasis.

**Figure 4:**
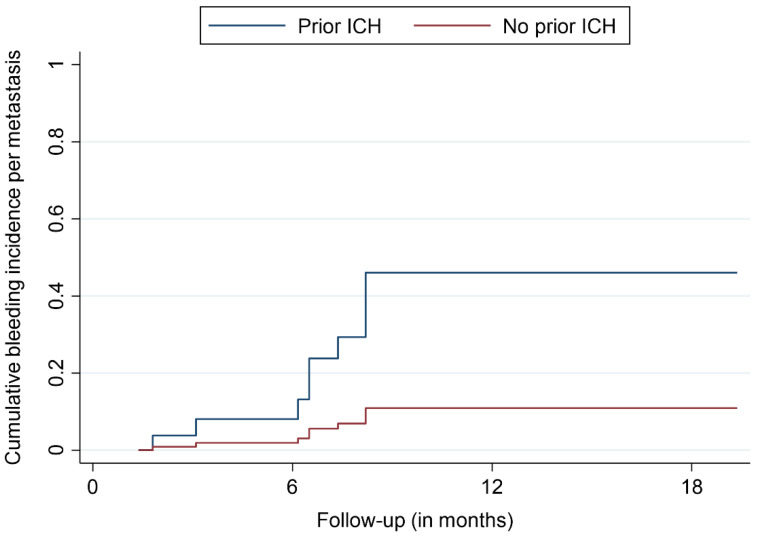
Cumulative incidence of bleeding events per metastasis, stratified by pretreatment intracranial hemorrhage (ICH).

**Figure 5:**
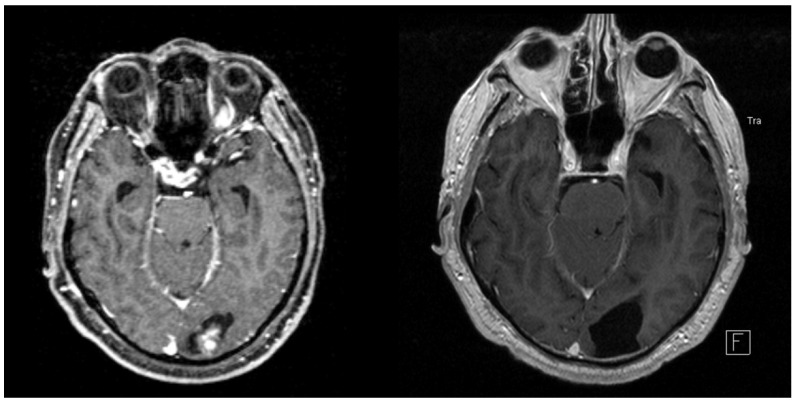
Contrast-enhanced magnetic resonance imaging (MRI) scans in the axial plane of a 72-year-old man suffering from a metastasized malignant melanoma. He underwent stereotactic radiosurgery (SRS) for an occipital lesion while receiving therapeutic anticoagulant therapy (ACT) with low-molecular-weight heparin (LMWH) due to a previously diagnosed pulmonary embolism (PE). Two months after treatment, a first imaging follow-up revealed asymptomatic intracranial hemorrhage (ICH) (**left**). At the last available follow-up, 25 months after SRS, the ICH was completely absorbed (**right**).

The median and mean follow-up times were 8.2 and 15.5 months, respectively. The OS rates were 66.8%, 44.0%, and 38.5% after 12, 24, and 36 months, respectively (Figure 1). At the last respective follow-up, 18 patients (44%) had died. Nine metastases (9%) in a group of five patients (12%) showed signs of ICH at the site of SRS treatment during MRI follow-up. The bleeding metastases were mostly localized in the frontal lobe (5/9 BM, 55%) and had a median size of 0.96 cc prior to SRS. The median age of the affected patients was 77.2 years. None of the affected patients showed neurological or other types of symptoms, with the diagnoses of bleeding BM only being made with MRI. The time to bleeding after SRS ranged between 1.8 and 8.2 months, with a mean time of 6.2 months. Three of the bleeding BM bled before SRS, with one such metastasis having previously received radiosurgical treatment. No ICHs were identified outside the irradiated brain volume. The bleeding incidences per patient were 1.9%, 5.3%, and 5.3% after 6, 12, and 18 months, respectively (Figure 2). The incidences of bleeding events per lesion were 2.1%, 12.4%, and 12.4% after 6, 12, and 18 months, respectively (Figure 3). The cumulative incidences of ICH stratified by previous bleeding events are shown in Figure 4. All of the observed ICHs occurred within 12 months after SRS. Several patient, treatment, and tumor characteristics were investigated to determine their impact on the bleeding occurrence. A confirmed bleeding event before SRS and BM from malignant melanomas were found to be significantly associated with an ICH after SRS (*p* = 0.02, *p* = 0.01). Previous ICH showed a subdistribution hazard ratio of 4.2 in the Fine and Gray competing risk regression model (*p* < 0.01, Figure 4). The cumulative bleeding incidences for such lesions were 8.0%, 46.1%, and 46.1% after 6, 12, and 18 months, respectively (Figure 4). Eight out of all nine (88%) bleeding BM originated from malignant melanomas, with six out of nine (66%) occurring in patients who were taking phenprocoumon as their ACT. Three of the eight lesions (38%) that bled before SRS showed radiographic signs of a new ICH after radiosurgery. An ICH was not significantly associated with a decreased survival time, and none of the observed deaths occurred due to an ICH. Figure 5 shows a representative case of an ICH after SRS.

## 4. Discussion

Herein, we report our bi-institutional experience of the ICH rates after SRS for BM. In general, ACT for patients with BM is complicated by two conflicting problems. First, these patients show an increased risk for the development of AF, VTE, DVT, and PE [27,29]. On the other hand, there seems to be a higher probability of ICHs in patients with BM who are receiving systemic anticoagulation treatments [31]. Applications of high doses of radiation energy—as typically used in SRS—might promote the risk of bleeding by interfering with the endothelial microenvironment and may trigger subsequent hemorrhage-inducing effects [18]. Our results indicated that SRS in patients receiving ACT did not lead to an increase in clinically relevant bleeding complications, either at the site of SRS or at other intracerebral locations. Moreover, the bleeding events seemed to occur relatively soon after SRS. Given the scarcity of available reports on this matter, we suggest that the use of ACT in these patients is safe. In general, a few studies and a meta-analysis found that there was no dramatically increased ICH risk for patients with BM receiving ACT [30,31,34,35,36,37,38]. However, some evidence of an increased ICH risk for BM in the case of patients receiving ACT is available as well [39]. The limited number of relevant studies suggests that the BM from certain histologies—such as renal cell carcinoma and malignant melanoma—tend to have an increased bleeding risk [30,31,34,35,37]. Similarly, in our study, nearly all the bleeding events occurred in patients who were suffering from malignant melanomas. Additionally, a previous bleeding event was more frequently noted for patients with a radiographic ICH after SRS [39]. However, the number of observed ICHs is too low to draw firm conclusions from these observations. Overall, potential ICH-modulating factors are mostly unknown and poorly understood given the lack of relevant data, especially concerning radiotherapy.

Horstman et al. reported on the ICH rates of 125 patients with BM regardless of the treatments used, and included cases with and without ACT [36]. Approximately 50% of these patients suffered from primary non-small-cell lung cancer (NSCLC), and a total of twelve ICHs occurred. Of these twelve bleeding events, eight occurred in patients with ACT, and this equated to a non-significant increase compared to the number of events in patients without ACT [36]. The authors found the highest rate of ICH in the patients undergoing WBRT and SRS as opposed to other treatments for BM [36]. The overall low incidence of bleeding events prevented the observation of any statistically significant findings [36]. Our results are supported by the data of Horstmann et al., with comparable frequencies of bleeding events [36]. Donato et al. conducted a matched cohort study with 293 patients suffering from BM, with one group receiving therapeutic enoxaparin, and the other group not receiving this treatment [31]. No difference in the ICH rates was observed, with the only significant risk factor of bleeding being metastatic disease caused by renal cell carcinoma or malignant melanoma (four-fold increase in risk compared to lung cancer), regardless of ACT [31]. Although 245 patients in the study received any kind of brain irradiation (SRS or WBRT), no subgroup analysis or further data on the influence of radiotherapy were provided [31].

Given the known effects of radiation on the cerebral vasculature, the lack of reports and data especially concerning SRS for BM is surprising [18]. However, there have been reports of vascular abnormalities and potentially associated ICH due to radiation [40,41,42]. These ICHs may occur after a significant delay of up to ten years after treatment and have been more commonly reported in pediatric patients [40,43,44]. Thus, we may see more bleeding events in patients with prolonged survival due to such vascular abnormalities. However, as BM still bear a dismal prognosis, the question remains whether such survival times can be realistically achieved.

In contrast to patients with BM, patients suffering from primary brain tumors, specifically glioma, have an increased risk of ICH when receiving ACT [34,45,46,47,48]. The overall bleeding rates for patients with from primary brain tumors ranged from 1.9 to 23% [34]. However, the rate of fatal ICHs in the available studies was less than 1%, as reported by Zwicker et al. [34]. This is in agreement with our findings in patients with BM receiving ACT. A fatal ICH seems to be a relatively rare event, especially given the scarcity of respective analyses and available data. In our study, this may be due to the patient cohort, as the BM sizes were relatively small. Moreover, the location of bleeding BM that can cause life-threatening complications may be another relevant factor. Herein, 88% of the metastases were located either in the frontal, parietal, or occipital lobes, or the cerebellum. Only two metastases in the pons and thalamus were treated. One would assume that larger BM in crucial neuroanatomical locations may be especially dangerous, leading to severe complications if bleeding occurs after (hypofractionated) SRS. Nevertheless, without any further data to analyze and discuss, the evidence on how to handle ACT for patients with BM undergoing SRS remains extremely limited.

In conclusion, we appraise the concomitant treatments of SRS and systemic anticoagulation and deemed them to be safe; therefore, we do not see a general need for the discontinuation of ACT in this clinical situation in the light of a limited overall survival time in this patient population. However, we are aware of the controversy of the subject, and the significant clinical impact ICHs might have, with ICHs often being adjunct to a significant functional decline and increased morbidity and mortality. The risk of ICH and use of ACT should be carefully assessed. Moreover, the specific drug for anticoagulation may be of relevance as well [49,50]. Our report is limited by the retrospective nature of the study and the small sample size. However, there is a profound lack of studies investigating the bleeding complications of BM patients treated with single-fraction SRS while receiving ACT. Thus, this analysis provides evidence for the relatively safe use of ACT in patients with small- and medium-sized BM treated with SRS. Finally, the risk for relevant ICH occurrence in the context of ACT and larger BM treated with (hypofractionated) SRS remains mostly unknown.

## 5. Conclusions

Patients receiving an ACT and single-fraction SRS for small- to medium-sized BM do not seem to have a clinically relevant risk of ICH. Previous bleeding and metastases originating from malignant melanomas may increase the risk of bleeding events after SRS. Further studies are necessary to validate our reported findings.

## Figures and Tables

**Table 1:** Patient and treatment characteristics.

Parameter	All Patients (*n* = 41, with *n* = 97 BM)
Age (y), median (range)	69.0 (32.6–84.4)
Sex, *n* (%)	
Male	23 (56)
Female	18 (44)
Performance status, *n* (%)	
ECOG 0	20 (49)
ECOG 1	16 (39)
ECOG 2	5 (12)
Reason for ACT, *n* (%)	
Pulmonary embolism	17 (41)
Atrial fibrillation	14 (34)
Deep-vein thrombosis	3 (7)
ACT, *n* (%)	
Phenprocoumon	15 (37)
Novel oral anticoagulants	13 (32)
Low-molecular-weight heparin	8 (20)
Number of treated BM, *n* (%)	
1	25 (61)
2	6 (15)
≥3	10 (24)
Tumor entity, *n* (%)	
Lung (NSCLC and SCLC)	20 (49)
Malignant melanoma	6 (15)
Renal cell	5 (12)
Breast	3 (7)
Colorectal	2 (5)
Other	5 (12)
BM with prior surgery, *n* (%)	3 (3)
BM with prior WBRT, *n* (%)	12 (12)
BM with prior SRS, *n* (%)	6 (6)
BM with prior conventional radiotherapy, *n* (%)	2 (2)
BM with prior bleeding, *n* (%)	8 (8)
Follow-up (months), median (mean, range)	8.2 (15.5, 1.7–77.5)
BM with ICH during follow-up, *n* (%)	9 (9)
BM size (cc), median (mean, range)	0.47 (0.02–10.28)
Prescription dose (Gy), median (range)	20 (16–22)
Maximum tumor dose (Gy), median (range)	29.2 (22.8–35.0)
Mean tumor dose (Gy), median (range)	24.8 (18.9–28.8)
Minimum tumor dose (Gy), median (range)	19.5 (12.5–30.0)

Abbreviations: *n* = number; BM = brain metastasis; y = years; ECOG = Eastern Cooperative Oncology Group performance status; ACT = anticoagulant therapy; NSCLC = non-small-cell lung cancer; SCLC = small cell lung cancer; WBRT = whole-brain radiotherapy; cc = cubic centimeters.

## Data Availability

The data that support the findings of this study are available from the corresponding author, F.E., upon reasonable request.
